# Craniofacial morphology in patients with obstructive sleep apnea: cephalometric evaluation

**DOI:** 10.1016/j.bjorl.2020.05.026

**Published:** 2020-07-18

**Authors:** Michele Tepedino, Gaetano Illuzzi, Michele Laurenziello, Letizia Perillo, Anna Maria Taurino, Michele Cassano, Laura Guida, Giuseppe Burlon, Domenico Ciavarella

**Affiliations:** a University of L’Aquila, Department of Biotechnological and Applied Clinical Sciences, L’Aquila, Italy; b University of Foggia, Department of Clinical and Experimental Medicine, Foggia, Italy; c Second University of Naples, Multidisciplinary Department of Medical-Surgical and Dental Specialties, Naples, Italy

**Keywords:** OSAs, Airway, Polysomnography, AHI, SO_2_, Nadir

## Abstract

**Introduction:**

Obstructive sleep apnea is characterized by a reduced airflow through the upper airways during sleep. Two forms of obstructive sleep apnea are described: the central form and the obstructive form. The obstructive form is related to many factors, such as the craniofacial morphology.

**Objective:**

To evaluate the correlation between the morphology of the cranial base, of the mandible and the maxilla, and obstructive sleep apnea severity.

**Methods:**

Eighty-four patients, mean age of 50.4 years old; 73 males and 11 females with obstructive sleep apnea were enrolled in the present study. Patients with high body mass index and comorbidities were excluded. Lateral cephalograms and polysomnography were collected for each patient to evaluate the correlation between craniofacial morphology and obstructive sleep apnea severity. A Spearman’s rho correlation test between cephalometric measurements and obstructive sleep apnea indexes was computed. Statistical significance was set at *p* < 0.05.

**Results:**

Patients with a severe obstructive sleep apnea presented a reduction of sagittal growth of both effective mandibular length and cranio-basal length. The mandibular length was the only variable with a statistical correlation with apnea-hypopnea index. Vertical dimension showed a weak correlation with the severity of obstructive sleep apnea. No correlation with maxillary sagittal dimension was shown.

**Conclusion:**

Obstructive sleep apnea severity may be correlated to mandibular and cranial base growth. Facial vertical dimension had no correlation with obstructive sleep apnea severity.

## Introduction

Obstructive Sleep Apnea (OSA) is characterized by the limitation of the passage of air through the upper airways.^1^, ^2^, ^3^ In adults, OSA is more prevalent in males than in females, with a frequency of 2:1. Smoking, obesity, increased neck circumference, tongue dimension and craniofacial malformations are the common condition associated with OSA.^4^, ^5^ In children, OSA may be associated to adenotonsillary hypertrophy.^6^ OSA is associated with many diseases or disorders such as: cardiovascular diseases,^7^ metabolic disorders (i.e., diabetes),^8^ gastric disorders (i.e., gastroesophageal reflux disease),^9^ respiratory disorders (i.e., asthma),^10^ emotional and psychological disorders^11^ and increased mortality rates.^12^

Cardio-respiratory evaluation and polysomnography are the instrumental evaluations used for OSA screening and diagnosis.^13^ In particular cases, patients can be evaluated with Drug-Induced Sleep Endoscopy (DISE).^14^ This type of evaluation may support the clinician in selecting the most appropriate treatment modality in cases with a diagnosis of OSA.

The influence of craniofacial morphology in the pathogenesis of OSA is controversial.^15^ Cephalometric evaluation was used in the description of the common facial form of patients with OSA.^16^ It described how the cervical, hyoid and mandibular position may influence the severity of OSA.^17^

The aim of the present study was to determine if the length and the vertical position of the anterior cranial base, the maxillary and the mandibular plane are correlated with polysomnography indexes of OSA severity. The null hypothesis was that no correlation exists between craniofacial morphology and OSA severity.

## Methods

This study was conducted following the Strengthening The Reporting of OBservational Studies in Epidemiology (STROBE) guidelines for observational studies.

The records of patients referred to the Orthodontics section at Univerisity of Foggia from January 2014 and September 2018, and having a diagnosis of OSA, were screened for the following inclusion criteria: age between 30 and 60 years old, assessment of daytime sleepiness through Epworth Sleepiness Scale (ESS) questionnaire, absence of tobacco use, Body Mass Index (BMI) lower than 34 kg/m^2^, no previous maxillo-facial or upper airway surgical treatment, no fixed oral appliance or mobile prosthetic rehabilitation, no previous CPAP treatment, no temporomandibular joint disease, diagnostic records including a lateral cephalogram.

Sample size calculation (G*Power version 3.1.9.2, Franz Faul, Universität Kiel, Germany) revealed that in order to be able to detect a medium effect size of 0.3^18^ with a I type error of 0.05 and a power of 0.85, a number of 75 subjects would be needed.

The procedures followed were in accordance with the Helsinki Declaration of 1975, as revised in 2008, and were approved by the Internal Review Board of the University of Foggia (Approval nº 43/CE/2019). The records were retrieved retrospectively, were analyzed anonymously, and patients signed a written informed consent to participate to future research at the time that the records were taken.

### Instrumental evaluation

**Figure 1:**
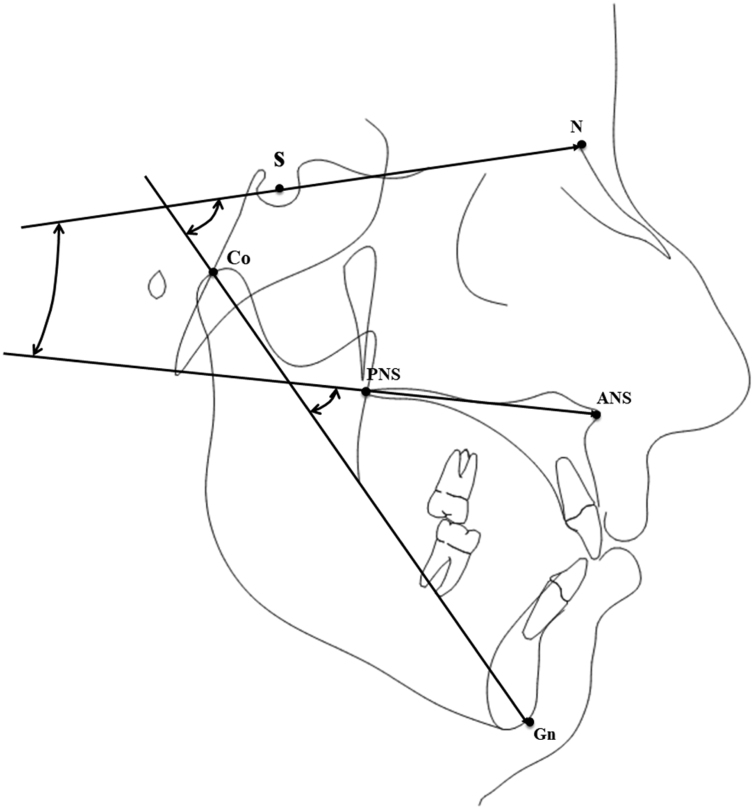
Tracing with the cephalometric variables used for the present study. S, Sella point; N, Nasion point; ANS, Anterior Nasal Spine; PNS, Posterior Nasal Spine; Co, Condylion point; Gn, Gnathion point.

Tracings were performed on lateral cephalograms by a blinded operator to collect the measurements shown in Fig. 1 and described in Table 1, comprising sagittal linear measurements and vertical angular measurements of the anterior cranial base, the maxillary plane, and the mandible. To evaluate the error of the method, 20 cephalograms were selected randomly and tracings were repeated 30 days later by the same examiner (M.T.). An Intra-Class Correlation (ICC) coefficient was calculated between the two set of measurements to evaluate the intra-operator reliability.

**Table 1:** Description of the evaluated cephalometric variables.

Cephalometrics evaluation	Description
N-Ba (mm)	The distance between Nasion (anterior part of nasal bone) and Basion (the postero-inferior part of Sfenoid bone)
Co-GN (mm)	The distance between Condilion (upper part of mandibular condyle) and Gnation (the anterion part of mandibular symphysis)
ANS-PNS (mm)	The distance between SNA (anterior nasal spine) and PNS (postero-nasal spine)
MXL-BL°	The angle between the MXL (maxillary line) and BL (anterior cranial base line)
MXL-Co-Gn°	The angle between MXL (maxillary line) and CO-GN line (the condilion-gnation line)
BL-CoGn°	The angle between the BL line (anterior cranial base line) and the CO-GN line (the condilion-gnation line)

Each patient received a Split Night Polysomnography (SN-PSG) and the first three hours of sleep were used for scoring, as recommended by several authors.^19^, ^20^, ^21^, ^22^ The registration was considered effective if the device successfully registered for more than 4 h without interruption. The data from the SN-PSG recording were used for a manual scoring according to the American Academy of Sleep Medicine (AASM) criteria from 2007. All subjects were evaluated for one night in a Sleep Laboratory using a type 2 portable device, the Embletta system X-100 (Flaga, Reykjavik, Iceland). The SN-PSG included Electroencephalographic (EEG), Electro-oculograms (EOG), Electromyograms (EMG), pulse oximetry channels, nasal cannulas, thoracic and abdominal respiratory effort bands, and body position sensors. Airflow was monitored by a nasal cannula and by oral thermistor. The thoracic-abdominal movements of all subjects were detected through two piezoelectric belts. Overnight continuous recordings of oxygen saturation were obtained by finger pulse oximetry. Snoring was recorded by a microphone placed at the neck, and note was taken of ECG findings and sleep position. Recording was performed after one night of adaptation to the hospital setting.

The data that were extracted from the SN-PSG recordings are described in detail in Table 2.

**Table 2:** Description of indexes recorded during an overnight polysomnography.

Polysomnographic evaluation	Description
AHI	Apnea-hypopnea index: the number of apnea and hypopnea events per hour of sleep.
SO2	Fraction of oxygen-saturated hemoglobin relative to total hemoglobin (unsaturated + saturated) in the blood.
NADIR	The lowest value of blood oxygen saturation registered overnight.

### Statistical analysis

Data have been analyzed using GraphPad Prism software 6.0 (GraphPad Prism Software, San Diego, CA, USA). Descriptive statistics were calculated, then data distribution was assessed by a Kolmogorov-Smirnov test and probability plots. To evaluate the correlation between SN-PSG data and craniofacial measurements, a Pearson’s correlation coefficient or a Spearman’s rho correlation was used depending on data distribution. First-type error was set as *p* < 0.05.

## Results

Eighty-four patients (mean age 50.4 years old; 73 males and 11 females) with OSA were enrolled in the present study.

Regarding the error of the method, the calculated ICC coefficient was excellent (> 0.85) for all the variables, revealing a good intra-observer reliability of the measurements.

Descriptive statistics for craniofacial measurements and SN-PSG indexes are reported in Table 3. The variables AHI, cranial base length (N-BA) and mandibular length (Co-Gn) showed a large Standard Deviation (N-BA: SD = 13.44 mm; Co-Gn: SD = 27.17 mm; AHI: SD = 20.21 mm).

**Table 3:** Descriptive statistics and normality test for cephalometric and polysomnographic variabes (n = 84).

	Mean	SD	SE	Normality test
N-BA (mm)	122.3	13.4	1.5	N.S.
Co-Gn (mm)	145.4	27.2	2.9	N.S.
SNA-SNP (mm)	61.59	6.6	0.7	< 0.05
MXL-BL°	24.93	5.6	0.6	< 0.05
MXL-Co-Gn°	49.85	5.3	0.6	N.S.
BL-Co-Gn°	74.78	6.4	0.7	< 0.05
AHI	37.02	20.2	2.2	<0.05
SaO2	93.12	2.9	0.3	N.S.
Nadir SaO2	77.48	10.4	1.1	N.S.

Because the majority of the variables were not normally distributed, a non-parametric statistical test was used. Spearman’ rho test (Table 4) detected a significant negative correlation between AHI and Co-Gn length (*p* <  0.001; *ρ* = −0.37) and between AHI and N-Ba (*p* <  0.05; ρ = −0.25). The maxillary length (ANS_PNS) showed a negative correlation with the Nadir (*p* <  0.01; *ρ* = −0.30), and a strong and significative positive correlation with N-BA (*p* <  0.001; *ρ* = 0.58) and Co-Gn (*p* <  0.001; *ρ* = 0.47).

**Table 4:** Spearman's rho correlation between the craniofacial variables and the polysomnography indexes (n = 84).

	N-BA (mm)	Co-Gn (mm)	SNA-SNP (mm)	MXL-BL°	MXL-Co-Gn°	BL-Co-Gn°	AHI	SaO_2_	Nadir SaO_2_
N-BA (mm)	−	0.701^b^ (<0.001)	0.581^b^ (<0.001)	−0.029 (0.792)	−0.162 (0.142)	−0.071 (0.522)	−0.254^a^ (0.020)	0.027 (0.806)	0.129 (0.242)
Co-Gn (mm)	0.701^b^ (<0.001)	−	0.475^b^ (<0.001)	−0.387^b^ (<0.001)	−0.076 (0.493)	−0.355^b^ (<0.001)	−0.371^b^ (<0.001)	0.191 (0.082)	0.069 (0.533)
SNA-SNP (mm)	0.581^b^ (<0.001)	0.475^b^ (<0.001)	−	−0.378^b^ (<0.001)	−0.164 (0.136)	−0.395^b^ (<0.001)	−0.107 (0.334)	0.004 (0.967)	0.127 (0.249)
MXL-BL°	−0.029 (0.792)	−0.387^b^ (<0.001)	−0.378^b^ (<0.001)	−	−0.309^b^ (0.004)	0.554^b^ (<0.001)	0.110 (0.318)	−0.171 (0.120)	−0.064 (0.560)
MXL-Co-Gn°	−0.162 (0.142)	−0.076 (0.493)	−0.164 (0.136)	−0.309^b^ (0.004)	−	0.574^b^ (<0.001)	0.163 (0.138)	−0.056 (0.613)	−0.080 (0.469)
BL-Co-Gn°	−0.071 (0.522)	−0.355^b^ (<0.001)	−0.395^b^ (<0.001)	0.554^b^ (<0.001)	0.574^b^ (<0.001)	−	0.234^a^ (0.032)	−0.246^a^ (0.024)	−0.030 (0.784)
AHI	−0.254^a^ (0.020)	−0.371^b^ (<0.001)	−0.107 (0.334)	0.110 (0.318)	0.163 (0.138)	0.234^a^ (0.032)	−	−0.470^b^ (<0.001)	−0.571^b^ (<0.001)
SaO2	0.027 (0.806)	0.191 (0.082)	0.004 (0.967)	−0.171 (0.120)	−0.056 (0.613)	−0.246^a^ (0.024)	−0.470^b^ (<0.001)	−	0.205 (0.061)
Nadir SaO2	0.129 (0.242)	0.069 (0.533)	0.127 (0.249)	−0.171 (0.120)	−0.080 (0.469)	−0.030 (0.784)	−0.571^b^ (<0.001)	0.205 (0.061)	−

a Statistically significant with *p* < 0.05.

b Statistically significant with *p* < 0.01.

The craniofacial divergence pattern of OSA patients was described by the MXL-BL, MXL-CoGn, and BL-CoGn angular variables, which presented a similar data dispersion (MXL-BL°: SD = 5.6; MXL-CoGn°: SD = 6.4; Bl-CoGn°: SD = 6.4).

Spearman’s rho test showed a positive correlation between the amount of mandibular rotation and AHI severity (*p* < 0.05, *ρ* = 0.23). The BL-CoGn° was positively correlated to SO2 (*p* < 0.05; *ρ* = −0.24). No correlation was found between the maxillary rotation and all the SN-PSG data (Table 4).

## Discussion

In the present study, the relationship between OSA severity and the linear dimension or vertical rotation of the main craniofacial structures related to the obstructive form of OSA (i.e., the anterior cranial base, the mandible, and the maxilla) was studied.

The research for cephalometric predictors in OSA patients is one of the most important topics in sleep medicine,^23^, ^24^ as well as the research of early predictive factors – like craniofacial morphology − in children that may be associated with OSA pathogenesis.^25^

The upper airway obstruction may be related to a collapse of soft tissue structures like the soft palate,^26^ to the position and/or dimension of the tongue,^27^ or to an altered position and/or dimension of the maxilla or the mandible.^28^, ^29^, ^30^

Craniofacial soft tissue evaluation is another important aspect.^31^ Many authors evaluated OSA patients by using photographic evaluation or the 3D surface scanning of the face.^32^, ^33^ Lee et al.^32^ suggested that patients with OSA showed a wider and flatter mid-third and lower-third of the face. In addition, the authors showed a reduction of maxillary and mandibular length. Tyan et al. showed a significant correlation between craniofacial measurements (i.e., cervico-mental contour ratio, Face-width ratio, tragion-ramus-stomion angle) and AHI in OSA patients.^31^ Liu et al. described the craniofacial and cephalometric parameters observed in OSA patients, and their influence in OSA treatment with mandibular advancement devices.^34^ The authors suggested two types of craniofacial and soft tissue shape of OSA patients: subjects with a forward maxilla, a small oropharynx, less erupted maxillary molars, small incisor overjet, and a small soft palate, and patients with maxilla retrusion, a larger oropharynx, a larger soft palate, and over-erupted maxillary molars. The first group of patients had an OSA related to the position of hard tissues; the second group had an OSA related to an increased volume of the soft tissues secondary to an increase of BMI.

In the present study, only patients with a normal BMI were evaluated to avoid the influence of a possible confounder, since the relationship between OSA and BMI variation is well described in the literature.^35^

Many studies evaluated the cranio-cervical position as a possible factor in OSA pathogenesis.^36^ Variation of head and cervical vertebrae position may change the pharyngeal space,^37^ and many authors reported that head posture resulted in significant differences in patients with OSA, compared to controls; in fact, an increase of the cranio-cervical angles induced an extended natural head position that reduces the airway obstruction.

In the present study, the main craniofacial structures that define the nasal and oral pharyngeal perimeter were evaluated: the cranial base length (N-Ba), the effective mandibular length (Co-Gn), the maxillary length (ANS-PNS), and the vertical rotation of such structures.

The results of the present study showed a significative negative correlation between the total mandibular length (measured from the top of the condyle to the lower-anterior part of the mandible) and AHI (*ρ* = −0.37; *p* < 0.001). The cranial base length showed a weak negative correlation with AHI (*ρ* = −0.25; *p* < 0.05) but showed a strong and highly significant correlation with the mandibular length (*ρ* = 0.70; *p* < 0.0001). It seems that mandibular growth may influence the OSA severity, and that the growth and/or position of the mandible is influenced by the length of the cranial base. There are contradicting reports in the literature about the influence of the cranial base growth and the development of malocclusions, and more specifically the growth of the mandible. A systematic review outlined the possibility of such association, and the results of the present study seem to confirm this observation.^38^ This finding is meaningful because the cephalometric assessment of the cranial base, such as for an orthodontic diagnosis, could be used to evaluate the predictable mandibular growth and the consequent risk of OSA development.

The maxillary length was not correlated with SN-PSG parameters, but showed a significant correlation with the length of the cranial base and the length of the mandible (Table 4).

Regarding the vertical rotation of the craniofacial structures, three parameters were evaluated: the vertical rotation of the mid-third of the face (MXL-BL°, the rotation of the maxillary plane in respect to the cranial base), the vertical rotation of the lower-third of the face (MXL-Co-Gn°, the rotation of the mandible in respect to the maxillary plane), and the overall cranial vertical rotation (BL-Co-Gn, the rotation of the mandible in respect to the cranial base).

The mid-third vertical rotation and the lower-third vertical rotation had no correlation with the SN-PSG indexes. The total cranial vertical rotation showed a weak positive correlation with the AHI (*ρ* = 0.23; *p* < 0.05) and a weak negative correlation with the SO2 (*ρ* = −0.24; *p* <  0.05). In addition, the cranial vertical rotation showed a significant negative correlation with the effective mandibular length (*ρ* = −0.35; *p* < 0.0001), and was highly correlated with the mid-third vertical rotation (*ρ* = 0.55; *p* < 0.0001) and the lower-third vertical rotation (*ρ* = 0.57; *p* < 0.0001). Regarding the limitations of the present study, the retrospective design and the use of bidimensional images should be underlined. However, care was taken to avoid any selection bias by using a rigid chronological order during sample retrieval and blinding the operator who made the tracings. In addition, even if three-dimensional radiographs provide a greater number of information, the results of the present study still have a greater generalizability because lateral cephalograms are still the most used type of radiographic exam in everyday orthodontic practice.

## Conclusion

According to the results of the present study, the obstructive forms of OSA may be related to an altered positional relationship between craniofacial structures. In particular, it can be concluded that:

A reduced Co-Gn mandibular length may influence the severity of OSA.

The mandibular length is highly correlated with the sagittal length of the cranial base;

An increased vertical rotation of the entire craniofacial complex showed a weak correlation with the AHI value.

## Conflicts of interest

The authors declare no conflicts of interest.
